# LAFS: A Fast, Differentiable Approach to Feature Selection Using Learnable Attention

**DOI:** 10.3390/e28010020

**Published:** 2025-12-24

**Authors:** Hıncal Topçuoğlu, Atıf Evren, Elif Tuna, Erhan Ustaoğlu

**Affiliations:** 1 Department of Statistics, Faculty of Sciences and Literature, Yildiz Technical University, 34210 Istanbul, Turkey; aevren@yildiz.edu.tr (A.E.); eozturk@yildiz.edu.tr (E.T.); 2 Department of Informatics, Faculty of Management, Marmara University, 34180 Istanbul, Turkey; erhan.ustaoglu@marmara.edu.tr

**Keywords:** feature selection, attention mechanism, information theory, deep learning, tabular data

## Abstract

Feature selection is a critical preprocessing step for mitigating the curse of dimensionality in machine learning. Existing methods present a difficult trade-off: filter methods are fast but often suboptimal as they evaluate features in isolation, while wrapper methods are powerful but computationally prohibitive due to their iterative nature. In this paper, we propose LAFS (Learnable Attention for Feature Selection), a novel, end-to-end differentiable framework that achieves the performance of wrapper methods at the speed of simpler models. LAFS employs a neural attention mechanism to learn a context-aware importance score for all features simultaneously in a single forward pass. To encourage the selection of a sparse and non-redundant feature subset, we introduce a novel hybrid loss function that combines the standard classification objective with an information-theoretic entropic regularizer on the attention weights. We validate our approach on real-world high-dimensional benchmark datasets. Our experiments demonstrate that LAFS successfully identifies complex feature interactions and handles multicollinearity. In general comparison, LAFS achieves very close and accurate results to state-of-the-art RFE-LGBM and embedded FSA methods. Our work establishes a new point on the accuracy-efficiency frontier, demonstrating that attention-based architectures provide a compatible solution to the feature selection problem.

## 1. Introduction

The rapid augmentation of high-dimensional data across scientific and industrial domains has made feature selection an indispensable component of the modern machine learning pipeline. In tasks ranging from genomic analysis to financial modeling, datasets can contain thousands or millions of features, encountering a phenomenon often termed the “curse of dimensionality” [1]. The presence of numerous irrelevant or redundant features can severely degrade the performance of predictive models, increase computational and storage costs, and hinder the interpretability of results [2]. Consequently, the development of algorithms that can efficiently and effectively identify a minimal, maximally informative subset of features remains a central challenge in machine learning research [3,4].

Existing feature selection methods can be broadly categorized into four families: filters, wrappers, embedded, and hybrid methods. The hybrid methods are combinations of these former three methods. Filter methods, such as those based on Mutual Information (MI) [5], are computationally efficient as they evaluate features based on their statistical properties, independent of any predictive model. On the other hand, wrapper and hybrid methods generally require a lot of computation time. However, this diversity is also their primary weakness; they assess features in isolation, rendering them “myopic” and often incapable of identifying feature subsets that are collectively powerful, especially in the presence of complex interactions [6].

For instance, wrapper methods, like Recursive Feature Elimination (RFE) [7,8], employ a predictive model to score and iteratively select feature subsets. This approach is powerful and often yields state-of-the-art results by directly optimizing for the performance of a specific classifier. This power, however, comes at a prohibitive computational cost, if it is used for specifying predictors. The iterative search, which involves training a model hundreds or thousands of times, becomes intractable for high-dimensional datasets. Embedded methods, such as L1 (Lasso) [9] regularization, integrate feature selection into the model training process itself. However, many embedded methods are inherently linear and may fail to capture non-linear feature dependencies.

We propose LAFS (Learnable Attention for Feature Selection), an end-to-end differentiable framework that learns to select features holistically. Instead of a greedy, sequential search, LAFS employs a neural attention mechanism [10] to assess all features in parallel and assign a context-aware importance score in a single forward pass. The core of our contribution is a hybrid, information-theoretic loss function designed to guide the learning process. By augmenting the standard task-based loss with an entropic regularizer on the attention distribution, we explicitly encourage the model to discover sparse and non-redundant feature subsets. Our work contributes to the growing body of research at the intersection of information theory and deep learning [11,12], demonstrating how classical information-theoretic principles can be leveraged to guide modern neural architectures.

Our contribution is threefold:

- A Novel Architecture: We propose LAFS, a fully differentiable attention-based model designed specifically for the task of feature selection.
- An Information-Theoretic Learning Objective: We introduce a hybrid loss function with an entropic regularizer that promotes the selection of minimal and non-redundant feature subsets.
- Comprehensive Empirical Validation: We demonstrate through a large-scale benchmark study across a diverse suite of datasets that our LAFS architecture matches the performance of the leading algorithms. Furthermore, on some datasets, including the highly multicollinear ones, it provides the real-world effectiveness of its entropic regularizer in handling feature redundancy.

## 2. Related Work

The challenge of feature selection has been a central theme in machine learning for decades, leading to a vast body of literature. Several comprehensive surveys are available that categorize the landscape of methods [13,14]. Our work, LAFS, positions itself at the intersection of classical feature selection principles and modern deep learning architectures.

### 2.1. Classical Feature Selection: Filters

Filter methods are computationally efficient and use statistical measures to rank features. Common criteria include Mutual Information (MI), the Chi-squared test [15], Fisher Score [16], and ReliefF [17], which estimates feature quality based on nearest-neighbor distances. Advanced filters like mRMR [18] go a step further by simultaneously maximizing feature relevance and minimizing inter-feature redundancy, a principle that shares a philosophical motivation with our entropic regularizer. However, these methods remain fundamentally limited by their inability to account for complex, multivariate feature interactions that a predictive model can capture.

### 2.2. Classical Feature Selection: Wrappers and Embedded Methods

Wrapper methods, such as Recursive Feature Elimination (RFE), directly optimize the feature subsets for modeling processes, often yielding superior performance. Their exhaustive, iterative nature, however, results in a combinatorial search that is computationally expensive for high-dimensional data. Some of the embedded methods offer a compromise by integrating the selection process into model training. An example, LASSO, which adds an L1 penalty to the loss function of a linear model, inducing sparsity in the feature coefficients.

Another notable embedded approach is “Feature Selection with Annealing (FSA)”, proposed by Barbu et al. [19]. This method integrates a deterministic annealing schedule into a constrained optimization framework. During training, the number of active features is gradually reduced from the total number of features, *p*, down to a target number, *k*. At each step, features with the smallest weights are pruned, effectively embedding a backward elimination process directly into the model’s training loop.

### 2.3. Hybrid Methods

To bridge the gap between the speed of filters and the power of wrappers, researchers have long explored hybrid methods. The typical approach involves a two-stage process: first, a fast filter method is used to pre-select a large subset of potentially relevant features, and second, a more computationally expensive wrapper or embedded method is applied to this reduced set. Early and influential examples include the work of Das [20], who proposed a boosting-based hybrid method.

A sophisticated example is the “Fuzzy Random Forest for feature selection (FRF-fs)” [21,22]. This algorithm employs a complex three-stage pipeline: (1) a Genetic Algorithm is used to find optimal fuzzy partitions for continuous features (a filter stage), (2) a Fuzzy Random Forest then ranks these features based on fuzzy information gain, and (3) a Sequential Forward Selection (SFS) wrapper is used to find the final subset. While powerful, these multi-stage approaches remain a sequence of not fully connected processes. Our LAFS framework can be viewed as a modern, differentiable reimagination of the hybrid concept, learning the “filtering” (via attention) and “wrapping” (via the classifier’s performance feedback) in a single, unified, end-to-end training process.

### 2.4. Attention and Transformers for Tabular Data

The unprecedented success of the Transformer architecture in natural language processing has inspired a new wave of research into its application for tabular data. TabNet [23] was a pioneering work in this area, utilizing a sequential attention mechanism to select features at each decision step. More recent works like the FT-Transformer [24] and SAINT [25] adapt the standard Transformer architecture for supervised learning. While these models are designed for high-accuracy classification, our LAFS model is explicitly architected as a feature selection tool, using its learned attention weights to output a final, reduced feature subset.

### 2.5. Information Theory in Deep Learning

Our work is grounded in the principles of information theory in deep learning. The Information Bottleneck (IB) principle [26] posits that an optimal model should learn a maximally compressed representation of the input that is still maximally informative about the target. This principle is widened through deep learning studies like variational information bottleneck [27]. Our entropic regularizer, H(w), can be viewed through this lens; by minimizing the entropy of the attention distribution, we are effectively creating an information bottleneck that compresses the input features into a sparse, informative context vector. Recent work has also explored using deep learning models, such as Deep InfoMax (DIM) [28], which focuses on maximizing mutual information, and other methods like autoencoders with a concrete distribution to perform differentiable feature selection [29,30].

## 3. Methodology: The LAFS Algorithm

In this section, we formally define the architecture and learning objective of the LAFS framework. Our goal is to create a single, end-to-end differentiable model that learns a sparse and informative feature subset by combining a non-linear attention mechanism with an information-theoretic loss function.

### 3.1. Problem Formulation

Given a dataset $D={\left\{\left(x_{i},y_{i}\right)\right\}}_{i=1}^{n}$, where each sample $x_{i}\in R^{d}$ is a vector of *d* features and $y_{i}$ is the corresponding class label, the goal of feature selection is to identify a subset of feature indices S⊂{1,…,d} with size |S|=k≪d, such that a model trained on the reduced feature vectors $x_{S}$ achieves optimal predictive performance.

### 3.2. Architecture

**Figure 1:**
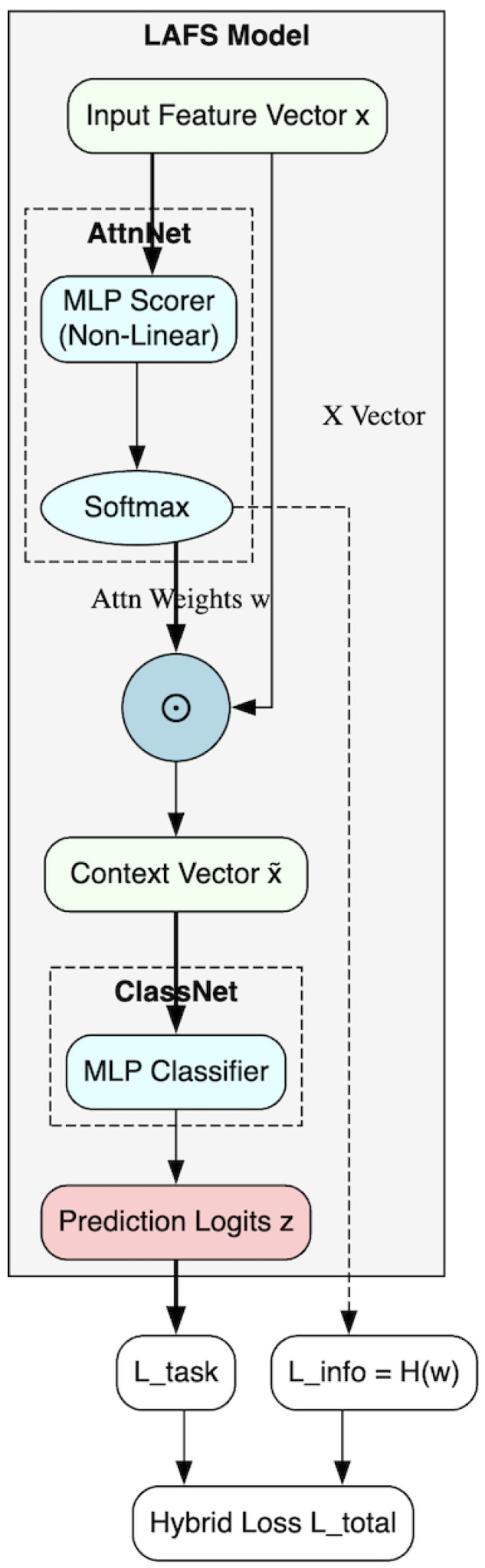
The LAFS architecture.

The LAFS model is composed of two primary sub-networks trained end-to-end: the Attention Scorer Network (AttnNet) and the Classifier Network (ClassNet). A schematic of the architecture is shown in Figure 1.

The input vector x is processed by the AttnNet to produce attention weights w. These weights are applied to x via element-wise multiplication (⊙) to create a context vector $\tilde{x}$, which is then fed to the ClassNet for prediction. Both the final logits z and the attention weights w are used in the hybrid loss function.

#### 3.2.1. The Attention Scorer Network (AttnNet)

The AttnNet is responsible for learning a dynamic, sample-specific feature importance mask. To capture potentially complex and non-linear relationships between features, we implement the AttnNet as a Multi-Layer Perceptron (MLP).

**Rationale:** A simple linear layered model can only learn linear combinations of features. By using an MLP with a non-linear activation function (e.g., ReLU), the AttnNet can model higher-order feature interactions, allowing it to understand that the importance of one feature might depend on the value of another. This expressive power is crucial for rivaling the performance of non-linear wrapper methods like RFE-LGBM.

For an input sample $x\in R^{d}$, the MLP-based AttnNet first projects the input into a hidden representation and then outputs a vector of pre-softmax scores, or logits, $s\in R^{d}$. These scores are then normalized using the softmax function to produce a probability distribution over the features, which we term the attention vector $w\in R^{d}$:

(1) $$w_{j}=\frac{\exp(s_{j})}{\sum_{l=1}^{d}\exp\left(s_{l}\right)}\;\mathrm{for}\;j=1,\ldots ,d$$

Each component $w_{j}$ represents the learned importance of the *j*-th feature for the given input sample x, with the property that $\sum_{j=1}^{d}w_{j}=1$.

**Potential Downside and Regularization:** The increased expressive power of an MLP comes with a higher number of parameters compared to a linear model, which can increase the risk of overfitting on datasets with very few training samples. This trade-off is managed through a combination of regularization techniques. Specifically, a Dropout [31] layer with a rate of ***p*** **= 0.5** is applied within the AttnNet’s MLP to prevent co-adaptation of neurons. Furthermore, we employ L2 regularization (Weight Decay) with a factor of **$1\times 10^{-4}$**, which is applied by the Adam optimizer [32] during the weight update step to penalize large weights. These techniques work in concert with our primary information-theoretic regularizer to ensure the model generalizes well.

#### 3.2.2. The Classifier Network (ClassNet)

The attention vector w is then used to re-weight the original feature vector x, producing a “context vector” $\tilde{x}$ that emphasizes the features deemed important:

(2) $$\tilde{x}=x⊙w$$

where ⊙ denotes element-wise multiplication. This context vector $\tilde{x}$ is then fed into the ClassNet, a standard multi-layer perceptron (MLP), to produce the final class prediction logits, z.

### 3.3. Hybrid Information-Theoretic Loss Function

The key to guiding LAFS to learn a sparse and effective feature mask lies in our custom loss function. The training process is a multi-objective optimization problem that simultaneously aims for high accuracy and high sparsity. This is formalized by our hybrid loss function, where the total loss is a weighted sum of a task-performance term and an information-theoretic regularization term:

(3) $$L_{\mathrm{total}}=L_{\mathrm{task}}+\lambda L_{\mathrm{info}}$$

The **task loss**, $L_{\mathrm{task}}$, is the standard cross-entropy loss. It ensures the model learns to be accurate by maximizing the log-probability of the correct class.

The **information-theoretic regularizer**, $L_{\mathrm{info}}$, is the Shannon entropy of the attention vector w. Minimizing this term encourages the model to produce low-entropy, “spiky” attention distributions, where a few features receive high weights and the rest receive near-zero weights, effectively promoting sparsity.

(4) $$L_{\mathrm{info}}=H\left(w\right)=-\sum_{j=1}^{d}w_{j}\log\left(w_{j}\right)$$

The hyperparameter λ is therefore the control parameter for the trade-off between these two competing objectives. The complete learning objective is to find the optimal model parameters $\Theta^{*}$ that solve the following:

(5) $$\Theta^{*}=\arg\min_{\Theta }E_{(x,y)∼D}\left[L_{\mathrm{task}}\left(p,y_{\mathrm{vec}}\right)-\lambda \sum_{j=1}^{d}w_{j}\log\left(w_{j}\right)\right]$$

where p is the softmax output of the ClassNet and w is the softmax output of the AttnNet. This formulation explicitly instructs the optimizer to find a solution that balances the need for an accurate feature representation with the structural constraint of a sparse and decisive attention mask.

### 3.4. Feature Importance Extraction

After training the model end-to-end over the entire training dataset, a global feature importance vector $I\in R^{d}$ is computed by averaging the sample-specific attention vectors $w^{\left(i\right)}$ for all samples $x_{i}$ in the training set:

(6) $$I_{j}=\frac{1}{n}\sum_{i=1}^{n}w_{j}^{\left(i\right)}$$

The final feature subset *S* is obtained by selecting the *k* features with the highest global importance scores $I_{j}$.

## 4. Experiments and Results

The first experiment is designed to test the algorithm’s ability to identify feature interactions. We generate a synthetic XOR dataset, which is a classic choice for benchmarking because it is linearly inseparable and forces feature selection methods to look for non-linear relationships or combinations of features. We also included a linearly separable dataset (SYNTH) experiment to test the performances of the methods where predictors are uniformly correlated.

As a second experiment, we conduct a comprehensive and a large-scale benchmark study for validating our method. This section details the experimental setup and presents the consolidated results across a diverse suite of datasets and feature sparsity levels.

### 4.1. Experiments on Synthetic XOR Dataset

To evaluate the algorithms’ ability to detect pure high-order interactions (where variables are individually useless but collectively predictive), we generated a 3-bit parity (XOR) dataset.

The data generation process for a sample *x* is defined as follows:

**Feature Generation:** We generate n=15 features from a standard normal distribution:

$$x∼N{\left(0,1\right)}^{15}$$

**Interaction Definition:** We designate the first three features ($x_{0},x_{1},x_{2}$) as “relevant”.

**Target Assignment:** The target class *y* is determined by the sign of the product of these three features:

$$y=I\left(\prod_{i=0}^{2}x_{i}>0\right)$$

where I(·) is the indicator function. The objective is to identify exactly $\left\{x_{0},x_{1},x_{2}\right\}$ (VDR target = 3/3).

### 4.2. Experiment Setup and Simulation Parameters on XOR Datasets

**Repetitions:** Each experiment was repeated 50 times with independent simulations for each sample size *n*.

**Evaluator:** The performance of selected features was evaluated using an XGBoost classifier (max_depth = 10, n_estimators = 100), chosen for its ability to model the XOR interaction.

### 4.3. LAFS Algorithm Configuration for XOR Datasets

The LAFS algorithm uses a neural network to learn feature importance masks. Given the difficulty of the XOR problem (which requires learning a precise three-way interaction with no marginal signal), we tuned the hyperparameters for stability:

**Epochs:** 300 (Increased from default 50 to allow convergence on complex loss landscape).

**Dropout:** 0.05 (Low dropout prevents breaking the fragile three-way interaction signal during training).

### 4.4. Benchmark Results for XOR Dataset

Table 1, Table 2, Table 3, Table 4 and Table 5 summarize the performance of each feature selection algorithm across increasing sample sizes (n∈{100,500,1000,2000,5000}).

#### 4.4.1. Results for *n* = 100 Samples

At extremely low sample sizes, no algorithm can reliably distinguish the complex three-way XOR signal from random noise. All methods perform close to random guessing (F1-Score ≈ 0.5).

#### 4.4.2. Results for *n* = 500 Samples

RFE-LGBM begins to detect the interaction signal significantly (VDR 2.12). LAFS also starts to learn (VDR 1.22), outperforming univariate filters like MI. Linear methods (FSA) and marginal filters (MI/mRMR) remain near random for *n* = 500.

#### 4.4.3. Results for *n* = 1000 Samples

With sufficient data, RFE-LGBM achieves perfect detection (VDR 3.00) and near-perfect downstream accuracy. LAFS improves further (VDR 2.16), confirming its ability to model non-linear interactions, though it requires more samples than gradient boosting trees to converge fully.

#### 4.4.4. Results for *n* = 2000 Samples

LAFS maintains solid detection (VDR∼2.18), proving it is superior to traditional filter methods for this problem class, though RFE-LGBM remains the most sample-efficient solver.

#### 4.4.5. Results for *n* = 5000 Samples

For very large sample sizes, results are consistent with theoretical expectations: univariate methods (MI, mRMR) and linear methods (FSA) never solve the problem (F1-Score remains random ≈ 0.5). RFE-LGBM remains perfect.

### 4.5. Synthetic Linear Data Generation

The synthetic dataset is generated using a linear threshold model with correlated features. The generation process for $n_{\mathrm{samples}}=1000$ and $n_{\mathrm{features}}=100$ is defined in the following two steps:

#### 4.5.1. Feature Generation: Uniform Correlation

To induce a uniform pairwise correlation between features, we construct the feature matrix *X* using a latent variable model. For each sample *i* and feature *j*, the following is calculated:

(7) $$\begin{array}{cc} \eta_{i,j}∼N(0,1) & (\mathrm{Independent}\;\mathrm{feature}\;\mathrm{noise}) \end{array}$$

(8) $$\begin{array}{cc} e_{i}∼N(0,1) & (\mathrm{Common}\;\mathrm{sample}\;\mathrm{noise}) \end{array}$$

(9) $$\begin{array}{cc} x_{i,j} & =\eta_{i,j}+e_{i} \end{array}$$

The common term $e_{i}$ introduces a covariance of 1 between any pair of features j≠k, resulting in a constant pairwise correlation of ρ=0.5.

#### 4.5.2. Target Generation: Linear Model

After generating the correlated feature matrix *X*, the target variable *y* is determined by a linear combination of these features. The coefficients (weights) are sampled from a continuous uniform distribution:

(10) $$\begin{array}{cc} w_{j}∼U\left(-1,1\right) & \mathrm{for}\;j=1,\ldots ,n_{\mathrm{features}} \end{array}$$

(11) $$\begin{array}{cc} s_{i} & =\sum_{j=1}^{n_{\mathrm{features}}}x_{i,j}w_{j} \end{array}$$

(12) $$\begin{array}{cc} y_{i} & =I(s_{i}>0) \end{array}$$

This process results in a linearly separable binary classification problem where all features are weakly relevant and uniformly correlated. Benchmark results of the methods for the linearly separable dataset (SYNTH) are given in Table 6.

### 4.6. Experiments with Benchmark Datasets

To ensure a robust and generalizable evaluation, we select a diverse suite of nine publicly available benchmark datasets, which are given in Table 7. These datasets are intentionally chosen from repositories such as PMLB and the original UCI archives to cover a wide range of characteristics, including “small *p*—small *n*”, “large *p*—small *n*”, and “large *p*—large *n*” scenarios.

**Benchmark Algorithms:** We compare our proposed architecture, **LAFS**, against five leading approaches that represent the primary families of feature selection: filter, wrapper, embedded, and hybrid methods.

- **MI (Mutual Information):** A classic, fast filter method.
- **mRMR (Minimum Redundancy Maximum Relevance):** An advanced filter considering feature redundancy.
- **RFE-LGBM (Recursive Feature Elimination with LightGBM):** The state-of-the-art wrapper method.
- **FSA (Feature Selection with Annealing):** An embedded method using a deterministic annealing schedule.
- **FRF-fs (Fuzzy Random Forest feature selection):** A sophisticated three-stage hybrid method.

**Evaluation Protocol:** For each of the nine datasets, we perform a 5-fold cross-validation. Within each fold, every algorithm is tasked with selecting features based on three distinct “*k*” values. This multi-level evaluation provides a comprehensive view of each algorithm’s performance across a range of sparsity pressures.

The quality of each selected feature subset is evaluated by training a downstream XGBoost classifier on the training portion of the fold and measuring its F1-Score and AUC on the held-out validation portion. Tested algorithms with three different “*k*” values, and the corresponding F1-Scores and AUCs are listed in Table 8.

Our proposed method, LAFS, firmly establishes itself as a promising method when the number of features (*p*) is small or medium, achieving compatible results with leading algorithms, “RFE-LGBM” and “FSA”. However, when the number of features is larger, as in the “Arcene” and “Gisette” datasets, LAFS fails to meet the results of other methods that can be considered as a limitation.

### 4.7. Future Work

This research opens several promising avenues for future investigation, turning the limitations we discovered into new research questions.

- **Taming Complex Architectures:** The failure of our Transformer-based models on tabular data is not an endpoint, but a challenge. Future work could focus on developing novel regularization techniques or self-supervised pre-training strategies specifically designed to control the high variance of self-attention mechanisms in low sample-to-feature ratio environments.
- **Data-Efficient Meta-Learning:** One may work on ‘LAFS-Meta‘-like architecture, which might be powerful on data-rich problems that can optimize parameters through the training process.
- **Extension to Other Domains:** The LAFS framework is inherently flexible. Extending the architecture to other domains is a natural next step. This includes adapting it for regression tasks by substituting the task loss with mean squared error and for survival analysis by incorporating a Cox proportional hazards loss, thereby broadening its applicability and impact.

## 5. Conclusions

This study aims to develop an innovative feature selection algorithm (LAFS), based on neural networks with an entropic loss function. LAFS is built upon a non-linear MLP-based attention mechanism specifically designed to model higher-order feature interactions. This capability is crucial for achieving high performance when the underlying data relationships are non-linear. Thus, LAFS offers a powerful, non-linear, and scalable solution alternative for feature selection.

LAFS is tested on synthetic and benchmark datasets against leading feature selection algorithms. The findings confirm that LAFS achieves its primary goal: A differentiable attention-based model designed specifically for the task of feature selection and the information-theoretic learning objective, which introduces a hybrid entropic loss function with a regularizer.

The results of synthetic and benchmark dataset experiments reveal that LAFS is a compatible feature selection algorithm. LAFS achieves the best or second-best performances with small and medium-sized feature datasets. However, our algorithm does not match the results of the leading algorithms, such as the RFE-LGBM wrapper and the embedded FSA, when the number of features is larger. Nevertheless, this can be improved by hyperparameter optimization.

Furthermore, our experiments provide a definitive, real-world validation of our core design principles. The consistent high performance of LAFS across a wide variety of datasets demonstrates the robustness of its MLP-based attention-gating mechanism, and it is capable of capture complex signals without being overwhelmed by noise. The success on datasets known for high multicollinearity (LSVT) also validates our information-theoretic entropic regularizer as an effective mechanism for achieving optimal feature selection.

## Figures and Tables

**Table 1:** Benchmark Results for *n* = 100.

Method	VDR	F1 Score	AUC
MI	0.6800	0.4798	0.5034
mRMR	0.7400	0.4617	0.4829
RFE-LGBM	0.5400	0.4735	0.4854
FSA	0.7800	0.4628	0.4843
FRF-fs	0.0200	0.0089	0.5012
LAFS	0.7400	0.4487	0.4747

**Table 2:** Benchmark Results for *n* = 500.

Method	VDR	F1 Score	AUC
MI	0.5600	0.4839	0.4816
mRMR	0.8200	0.4883	0.4913
RFE-LGBM	2.1200	0.8026	0.8219
FSA	0.6000	0.4973	0.4933
FRF-fs	0.0000	0.0000	0.5000
LAFS	1.2200	0.4911	0.4940

**Table 3:** Benchmark results for *n* = 1000.

Method	VDR	F1 Score	AUC
MI	0.4400	0.4982	0.4990
mRMR	0.5600	0.5068	0.4995
RFE-LGBM	3.0000	0.9786	0.9974
FSA	0.5000	0.4995	0.4954
FRF-fs	0.0000	0.0000	0.5000
LAFS	2.1600	0.6858	0.6912

**Table 4:** Benchmark results for *n* = 2000.

Method	VDR	F1 Score	AUC
MI	0.5200	0.5016	0.5000
mRMR	0.7600	0.4932	0.4960
RFE-LGBM	3.0000	0.9910	0.9994
FSA	0.5800	0.5117	0.5081
FRF-fs	0.0000	0.0000	0.5000
LAFS	2.1800	0.6542	0.6526

**Table 5:** Benchmark results for *n* = 5000.

Method	VDR	F1 Score	AUC
MI	0.6200	0.5161	0.5196
mRMR	0.6200	0.4962	0.4954
RFE-LGBM	2.9800	0.9853	0.9895
FSA	0.5600	0.4961	0.4970
FRF-fs	0.0000	0.0000	0.5000
LAFS	1.7800	0.5828	0.5824

**Table 6:** Benchmark results for SYNTH dataset at different *k* values.

		*k* = 10		*k* = 30		*k* = 50	
**Dataset**	**Algorithm**	**F1**	**AUC**	**F1**	**AUC**	**F1**	**AUC**
SYNTH	FRF-fs	0.0000	0.5000	0.0000	0.5000	0.0000	0.5000
	FSA	0.6681	0.7651	0.7149	0.7858	0.7180	0.8059
	LAFS	0.6814	0.7546	0.7200	0.7926	0.7544	0.8448
	MI	0.6764	0.7562	0.7052	0.7881	0.7306	0.8129
	RFE-LGBM	0.7082	0.7911	0.7703	0.8576	0.7634	0.8576
	mRMR	0.6693	0.7616	0.7095	0.7835	0.7238	0.8057

**Table 7:** Overview of the nine benchmark datasets used in the comprehensive evaluation. The table shows the total number of rows (*n*) and features (*p*) for each dataset, along with the specific number of features selected (*k*).

Dataset	Rows (*n*)	Features (*p*)	*k1*	*k2*	*k3*
zoo	101	16	3	5	10
spectf	267	44	3	5	10
australian	690	14	3	5	10
spambase	4601	57	10	30	50
tic_2000	9822	85	10	30	50
miniboone	130,065	50	10	30	50
lsvt	126	309	10	15	30
arcene	200	10,000	10	500	1000
gisette	7000	5000	10	250	500

**Table 8:** Detailed F1-scores and AUC scores for each algorithm across all benchmark datasets and sparsity levels (*k1*, *k2*, and *k3*).

					*k1*		*k2*		*k3*	
Dataset	Algorithm	*k1*	*k2*	*k3*	F1	AUC	F1	AUC	F1	AUC
ZOO	FRF-fs	3	5	10	0.5608	0.8705	0.7737	0.9532	0.8991	0.9867
	FSA	3	5	10	0.6672	0.9341	0.8261	0.9803	0.9325	0.9987
	LAFS	3	5	10	0.6898	0.9451	0.8982	0.9880	0.9418	0.9943
	MI	3	5	10	0.5626	0.8747	0.7866	0.9670	0.9537	0.9977
	RFE-LGBM	3	5	10	0.7107	0.9119	0.9120	0.9951	0.9129	0.9955
	mRMR	3	5	10	0.7308	0.9474	0.8730	0.9790	0.9057	0.9978
AUSTRALIAN	FRF-fs	3	5	10	0.8242	0.8467	0.8262	0.9126	0.7963	0.8731
	FSA	3	5	10	0.8504	0.9137	0.7957	0.9012	0.8426	0.9149
	LAFS	3	5	10	0.8191	0.9001	0.7892	0.8877	0.8293	0.9121
	MI	3	5	10	0.8201	0.9103	0.7962	0.9028	0.8484	0.9249
	RFE-LGBM	3	5	10	0.5395	0.6237	0.6605	0.7789	0.8487	0.9212
	mRMR	3	5	10	0.8329	0.9051	0.8032	0.9023	0.8462	0.9143
SPECTF	FRF-fs	3	5	10	0.0000	0.5000	0.0000	0.5000	0.0000	0.5000
	FSA	3	5	10	0.8936	0.9032	0.9157	0.9288	0.9280	0.9222
	LAFS	3	5	10	0.8882	0.8923	0.9252	0.9345	0.9311	0.9305
	MI	3	5	10	0.9005	0.9006	0.9178	0.9098	0.9309	0.9381
	RFE-LGBM	3	5	10	0.9106	0.9371	0.9346	0.9380	0.9276	0.9303
	mRMR	3	5	10	0.9039	0.8988	0.9145	0.9289	0.9364	0.9418
SPAMBASE	FRF-fs	10	30	50	0.6472	0.8371	0.7706	0.9089	0.7044	0.8787
	FSA	10	30	50	0.9047	0.9679	0.9315	0.9847	0.9380	0.9866
	LAFS	10	30	50	0.9026	0.9693	0.9227	0.9810	0.9370	0.9868
	MI	10	30	50	0.9105	0.9689	0.9307	0.9828	0.9392	0.9870
	RFE-LGBM	10	30	50	0.8844	0.9592	0.9346	0.9859	0.9409	0.9871
	mRMR	10	30	50	0.8999	0.9663	0.9314	0.9853	0.9379	0.9865
MINIBOONE	FRF-fs	10	30	50	0.0000	0.5000	0.0000	0.5000	0.0000	0.5000
	FSA	10	30	50	0.8316	0.9619	0.8903	0.9814	0.9010	0.9844
	LAFS	10	30	50	0.8358	0.9641	0.8736	0.9770	0.9010	0.9844
	MI	10	30	50	0.8385	0.9651	0.8758	0.9774	0.9010	0.9844
	RFE-LGBM	10	30	50	0.8724	0.9761	0.8971	0.9838	0.9010	0.9844
	mRMR	10	30	50	0.8405	0.9648	0.8912	0.9818	0.9010	0.9844
TIC_2000	FRF-fs	10	30	50	0.0000	0.5344	0.0000	0.5351	0.0033	0.5780
	FSA	10	30	50	0.0597	0.6993	0.1057	0.7119	0.0992	0.7069
	LAFS	10	30	50	0.0584	0.6349	0.0789	0.6723	0.1041	0.7055
	MI	10	30	50	0.0847	0.6973	0.0962	0.7045	0.1014	0.7091
	RFE-LGBM	10	30	50	0.0843	0.6424	0.0989	0.6951	0.1065	0.7072
	mRMR	10	30	50	0.0610	0.6983	0.0980	0.7143	0.0968	0.7058
LSVT	FRF-fs	10	15	30	0.8438	0.8431	0.8393	0.8108	0.8367	0.8469
	FSA	10	15	30	0.8646	0.8897	0.8669	0.8917	0.8722	0.8886
	LAFS	10	15	30	0.8619	0.8652	0.9065	0.8853	0.8988	0.9016
	MI	10	15	30	0.8202	0.8558	0.8365	0.8909	0.8551	0.8793
	RFE-LGBM	10	15	30	0.8720	0.8472	0.8809	0.8821	0.8984	0.9051
	mRMR	10	15	30	0.8619	0.8848	0.8577	0.9015	0.8687	0.9097
ARCENE	FRF-fs	10	500	1000	0.0211	0.5346	0.2801	0.5959	0.1283	0.5482
	FSA	10	500	1000	0.7190	0.8576	0.7209	0.8594	0.7190	0.8326
	LAFS	10	500	1000	0.6297	0.7799	0.7848	0.9039	0.6858	0.8402
	MI	10	500	1000	0.7562	0.8142	0.8037	0.9086	0.7725	0.9005
	RFE-LGBM	10	500	1000	0.6647	0.7968	0.7566	0.8701	0.7512	0.8787
	mRMR	10	500	1000	0.7078	0.8071	0.6957	0.8360	0.7461	0.8522
GISETTE	FRF-fs	10	250	500	0.0398	0.5103	0.0473	0.5105	0.1649	0.5101
	FSA	10	250	500	0.9291	0.9720	0.9787	0.9968	0.9811	0.9970
	LAFS	10	250	500	0.8933	0.9493	0.9643	0.9930	0.9685	0.9952
	MI	10	250	500	0.9074	0.9493	0.9763	0.9955	0.9773	0.9962
	RFE-LGBM	10	250	500	0.9152	0.9620	0.9797	0.9970	0.9806	0.9970
	mRMR	10	250	500	0.9171	0.9621	0.9751	0.9954	0.9779	0.9964

## Data Availability

The data that support the findings of this study are available from the corresponding author upon request.
